# Increasing agility and visibility in scientific publishing

**DOI:** 10.20945/2359-3997000000658

**Published:** 2023-05-10

**Authors:** Beatriz D. Schaan, Bruno Ferraz-de-Souza

**Affiliations:** 1 Universidade Federal do Rio Grande do Sul Porto Alegre RS Brasil Universidade Federal do Rio Grande do Sul, Porto Alegre, RS, Brasil. Hospital de Clínicas de Porto Alegre, Porto Alegre, RS, Brasil; Hospital de Clínicas de Porto Alegre Porto Alegre RS Brasil; 2 University of Notre Dame Australia School of Medicine Fremantle Australia School of Medicine, University of Notre Dame Australia, Fremantle, Australia

Since its inception, AE&M aimed to establish itself as a leading source of high-quality scientiﬁc information in the areas of endocrinology and metabolism ( 1 ). In that sense, maintaining open access to our articles was paramount to amplify the reach of such information in a globalized, albeit inequitable, world ( 2 ). Aiming to continue to serve the community of readers, authors, and reviewers in the best possible way, two new implementations are underway: AE&M has joined PubMed Central (PMC), and from May 2023, AE&M will adopt the continuous publication model.

AE&M's incorporation into PMC reﬂects its growth and scientiﬁc relevance in the ﬁeld. PMC is a free full-text repository of biomedical and life sciences journal literature at the U.S. National Institutes of Health's National Library of Medicine (NIH/NLM), directly linked to its preeminent search engine. Created in 2000, it houses more than 7.6 million records and, like SciELO, PMC-indexed journals make their issues and articles available in full format. Its global reach will certainly bring even more visibility and prominence to the research ﬁndings published in AE&M.

Another remarkable achievement is the adoption of the continuous publication model, beginning next month. This means that articles will be released online as soon as they are ready for publication, without the need to wait for other articles to compose a complete issue of the journal. This change aims to increase the efficiency of the publication process and to ensure that readers have faster access to important research ﬁndings in the areas of endocrinology and metabolism. We also believe that this change will beneﬁt our authors, providing greater visibility and recognition for their work.

With these changes, AE&M aligns itself with recent trends in scientiﬁc publishing to increase the agility in making research available for the community. We will keep the high standards of quality that are expected of AE&M, and we hope that these implementations will further enhance the experience for our readers and authors. These achievements result from the effort and commitment of the entire editorial team, to which we are immensely grateful. Our community is responsible for allowing AE&M to progress as a valuable and reputable source of information in our ﬁeld.
